# Th17 cytokines and factors modulating their activity in patients with pernicious anemia

**DOI:** 10.1007/s12026-023-09399-9

**Published:** 2023-06-03

**Authors:** Dariusz Kajdaniuk, Wanda Foltyn, Elżbieta Morawiec-Szymonik, Zenon Czuba, Ewa Szymonik, Beata Kos-Kudła, Bogdan Marek

**Affiliations:** 1 Department of Pathophysiology, Chair of Pathophysiology and Endocrinology, Medical University of Silesia, H. Jordana 19, 41-808 Zabrze, Katowice, Poland; 2 Department of Endocrinology and Neuroendocrine Tumors, Chair of Pathophysiology and Endocrinology, Medical University of Silesia, Katowice, Poland; 3 Department of Internal Medicine and Oncological Chemotherapy, Andrzej Mielęcki Independent Public Clinical Hospital, Katowice, Poland; 4 Department of Microbiology and Immunology, Medical University of Silesia, Katowice, Poland; 5 Department of Anesthesiology and Intensive Care, Stanislaw Szyszko Independent Public Clinical Hospital No. 1, Zabrze, Poland

**Keywords:** Th17, Cytokines, Transforming growth factor beta, TGF beta, Pernicious anemia, Autoimmune disease

## Abstract

The effects of specific cytokines produced by T cell subsets (such as Th1, Th2, and newly discovered Th17, Treg, Tfh, or Th22) are diverse, depending on interactions with other cytokines, distinct signaling pathways, phase of the disease, or etiological factor. The immunity equilibrium of the immune cells, such as the Th1/Th2, the Th17/Treg, and the Th17/Th1 balance is necessary for the maintenance of the immune homeostasis. If the balance of the T cells subsets is damaged, the autoimmune response becomes enhanced which leads to autoimmune diseases. Indeed, both the Th1/Th2 and the Th17/Treg dichotomies are involved in the pathomechanism of autoimmune diseases. The aim of the study was to determine the cytokines of Th17 lymphocytes as well as the factors modulating their activity in patients with pernicious anemia. The magnetic bead-based immunoassays used (Bio-Plex) allow simultaneous detection of multiple immune mediators from one serum sample. In our study, we showed that patients suffering from pernicious anemia develop the Th1/Th2 imbalance with a quantitative advantage of cytokines participating in Th1-related immune response, the Th17/Treg imbalance with a quantitative advantage of cytokines participating in Treg-related response, as well as the Th17/Th1 imbalance with a quantitative predominance of cytokines participating in Th1-related immune response. Our study results indicate that T lymphocytes and their specific cytokines play an role in the course of pernicious anemia. The observed changes may indicate the immune response to pernicious anemia or be an element of the pernicious anemia pathomechanism.

**Supplementary information:**

The online version contains supplementary material available at 10.1007/s12026-023-09399-9.

## Introduction

Pernicious Anemia (PA) is an autoimmune disease in the pathogenesis of which autoantibodies against the intrinsic factor and the gastric enzyme H^+^/K^+^-ATPase (proton pump) are involved [1–6]. PA is a serious sequelae of autoimmune gastritis (AIG) [2, 4, 5]. The role of *Helicobacter pylori* infection in activating AIG is still uncertain.

The pathomechanism of autoimmune diseases is based on cellular and/or humoral response. Both mechanisms can complement each other and penetrate each other, thus most often they occur simultaneously. In autoimmune diseases with predominance of the cell effector mechanisms (such as Hashimoto's thyroiditis, diabetes mellitus t.1), the initiating role is assigned to subtypes 1 and 17 of T helper (Th) lymphocytes, while the cytotoxic lymphocytes, neutrophils, macrophages, and their cytokines are responsible for the effector function. In autoimmune diseases with predominance of the humoral effector mechanisms (such as autohemolytic anemia or Graves' disease), the initiating role in stimulating B lymphocytes to produce autoantibodies is assigned to Th2 lymphocytes. Another classification of autoimmune diseases is based on the location of the autoantigen [7]. In organ-specific diseases (such as PA, Hashimoto's thyroiditis, Graves’ disease), the presence of the autoantigen is restricted to specific organs, tissues, or cells [7–9]. Regardless of the above-mentioned classifications, a non-specific inflammatory process can provide a "danger signal" accompanied by the release of the autoantigens as a result of tissue destruction. Chronic inflammatory diseases associated with autoimmune processes also include PA [7].

CD4 + T cells differentiate into various lineages of T helper cells, characterized by distinct developmental regulation and their function in the immune response. For years, it was thought that CD4 + Th cells divide into two lineages named Th1 and Th2. Eventually, it has been shown that Th lymphocytes can also differentiate into IL-17-producing cells independently of Th1 or Th2 development, thereby establishing Th17 cells as a unique T helper cell lineage [9, 10].

Th17 cells secrete several interleukins (IL), including IL-17A, IL-17F, IL-21 and IL-22 [10–13]. IL-17F shares the highest amino acid identity with IL-17A [13], and thus these two together can form a heterodimeric cytokine IL-17AF [14]. IL-17 family also includes IL-17B, IL-17C, IL-17D [13]. IL-17E (nowadays known as IL-25), first assigned to Th17, is in fact produced by Th2 cells [15]. Differentiation of naive T cells towards the Th17 phenotype is supported by several “differentiating cytokines” including IL-1β, IL-6, IL-21, IL-23, and Transforming Growth Factor-β (TGF-β) [13, 16–20]. Th17 cells express two orphan nuclear receptors, retinoic-acid-receptor-related orphan receptor-α (RORα) and RORγ, which are required for the Th17-cell development. The process is initiated by TGF-β and IL-6 [10, 20]. TGF-β prevents Th1 and Th2 differentiation by suppressing Signal Transducer, Activator of Transcription (STAT4) and GATA-3 expression, allowing Th17 development. However, Th17 differentiation also occurs in the absence of TGF-β signaling [13, 21, 22]. Th17-cell-specific genetic programming is also mediated by STAT3, downstream of IL-6 and IL-21 [10]. The differentiation is sustained by IL-21 [10, 23] which, secreted by Th17 cells, acts in an autocrine manner [13, 24]. IL-22, also produced by Th17 cells, acts in a similar way [10]. The Th17 development is most probably completed or maintained by IL-23 [10]. IL-1β has been shown to enhance Th17 responses in the presence of IL-23 [13, 17, 18]. Understanding the role of the cytokines involved in the induction of Th17 cells differentiation as well as the balance between Th17 versus Treg (T regulatory) and Th1 cells during development of autoimmunity, requires further investigation [13].

Th17 cells have been shown to play an important role in immune responses to infectious agents, as well as in various immune diseases. IL-17 are generally thought to be proinflammatory [10], which can be beneficial to the host during infection. However, an uncontrolled or inappropriate Th17 activation has been linked to several autoimmune pathologies [13]. Aberrant regulation of Th17 cells may play a role in the pathogenesis of inflammatory and autoimmune disorders [10, 25–28], as well as in host response to cancer [14, 28]. The IL-17/Th17-associated autoimmune diseases include inflammatory bowel disease, multiple sclerosis, psoriasis, ankylosing spondylitis, rheumatoid arthritis, Sjogren’s syndrome, and Takayasu arteritis [7, 14, 29–31]. High levels of IL-17 have been detected in the blood of patients with systemic lupus erythematosus, colitis ulcerosa, and Crohn disease [7, 32, 33]. Moreover, IL-17 neutralizing antibodies are currently used as an element of treatment strategies of some autoimmune diseases [7]. Evidence has recently been provided that gastric mucosa of PA patients harbour a high proportion (20%) of autoreactive activated CD4 + T-cell clones that specifically recognize intrinsic factor. Most of these clones (94%) showed a Th17 or Th1 profile. All intrinsic factor-specific clones also produced TNF-α and IL-21 [34].

The aim of the study was to determine cytokines of Th17 lymphocytes and factors modulating their activity in patients diagnosed with PA.

## Materials and methods

The study was approved by the Bioethical Committee of the Medical University of Silesia (No. KNW/0022/KB1/84/10). The procedures used in this study adhere to the tenets of the Declaration of Helsinki. The study group (PA) was made up of 34 people with a newly diagnosed autoimmune disease of the hematopoietic system, i.e. PA. The group comprised 23 women and 11 men aged 30 to 59 years old. The inclusion criteria were age over 18 years, the diagnosis of PA based on a typical clinical picture, as well as fulfillment of at least 2 of the following 3 diagnostic criteria: 1) a reduced serum level of vitamin B_12_ (below 200 pg/ml); 2) positive results for one or both intrinsic factor (IFAb) and gastric parietal cells (GPCAb) antibodies; 3) a positive therapeutic test, that is, an increase in the number of reticulocytes after 5–10 days (reticulocytic break), reduction of iron or LDH in the blood by 50% of baseline, remission of thrombocytopenia or/and neutropenia within 2 weeks, regression of anemia and hypersegmentation of granulocyte nuclei after 2–4 weeks following a single parenteral administration of 1000 μg of vitamin B_12_. Exclusion criteria involved malignancy, renal failure (eGFR < 45 ml/min./1.73m^2^), liver failure (bilirubin > 34,2 µmol/l), symptomatic circulatory insufficiency, symptomatic respiratory failure, severe neurological diseases, and mental disorders. The patients had no clinical signs of concomitant autoimmune disease, and the available test results did not suggest it either. Due to the widespread occurrence of autoimmune thyroid disease, we determined the antibodies against the thyroid peroxidase (TPOAb) and thyroglobulin (TgAb) and excluded from the study group positive patients. All patients had AIG with the presence of IFAb and/or GPCAb. The patients were free of *H. pylori* infection at study entry. If *H. pylori* infection was confirmed, successful eradication was performed before entering the study. At the time of enrolling patients, there was no indication for proton pump inhibitor therapy. The control group (C) comprised 25 people, 18 women and 7 men, matched for age. Criteria for inclusion were age over 18 years, exclusion of autoimmune disease based on the interview and available test results, negative titers of the intrinsic factor and gastric parietal cells antibodies, no clinical signs of organ dysfunction, no abnormalities in blood count or blood smear, no aberrations in basic laboratory parameters (listed below), including vitamin B_12_ serum concentration, no long-term pharmacotherapy, no history of autoimmune diseases in the family. In both groups, PA and C, blood samples were collected in the morning in fasting state from antecubital vein. Serum samples were stored at -75 °C [5, 6].

16 cytokines (IL-1β, IL-4, IL-6, IL-10, IL-17A, IL-17F, IL-21, IL-22, IL-23, IL-25, IL-31, IL-33, IFN-γ, TNF-α, sCD40L, TGF-β1) were analyzed simultaneously by the use of Bio-Rad Bio-Plex™ 200 System and commercial Bio-Plex Pro Human Cytokine Assay kits (Bio-Rad Laboratories, Inc.). These magnetic bead-based immunoassays allow simultaneous detection of multiple immune mediators from one sample, in a single well of a 96-well microplate. The samples were analyzed using the system flow cytometer with two lasers (532 nm Nd-Yag laser to excite phycoerythrin and a 635 nm solid state laser to excite dyes inside beads to determine ‘‘color’’ or ‘‘region’’) and associated optics to measure the different molecules bound to the surface of the beads. A high-speed digital signal processor in the system managed the fluorescent output. Immune mediators were calculated using Bio-Plex Manager™ Software and presented as concentration (pg/ml) [35–38]. Serum concentration of vitamin B_12_, iron, ferritin, folic acid, as well as peripheral blood count with smear, platelets count determined by the use of manual method, INR, APTT, PT, fibrinogen, D-dimers, proteinogram, total protein concentration, basic biochemical tests (creatinine, glucose, bilirubin, ALT, electrolytes, lipid profile, uric acid, CRP), TSH, FT4, FT3, TPOAb, TgAb were determined using routine kits of biochemical analysers. Autoantibodies against the IFAb and the GPCAb were determined by Enzyme-Linked Immunosorbent Assay (ELISA).

### Statistical methods and tools

The clinical database was created using a licensed version of EXCEL v. 2016 spreadsheet by Microsoft. Statistical calculations were performed by the use of licensed statistical packages: Statistica v. 7.1 PL by StatSoft, MedCalc Statistical Software v.14.10.2 (MedCalc Software bvba, Ostend, Belgium) and PQStat Software v. 1.6.6. In the statistical analysis, the level of significance (type I error) was adopted: p(α) < 0.05 and the following tests were used. The compliance of a given quantitative variable with the normal distribution was verified using the Shapiro–Wilk test (if the test result is < 0.05, the distribution deviates from the normal distribution). When comparing two groups, the hypothesis of the same level of a given quantitative variable with a normal distribution was verified by the parametric Student's t-test. This test was preceded by Fisher's test for two variances. In case of finding heterogeneity of variance, the Student's t-test was used for two means with unequal variances. The hypothesis about the same level of a given quantitative variable characterized by a distribution deviating from the normal when comparing the two groups was verified by the non-parametric Mann–Whitney *U* rank sum test. In the comparative analysis between groups, the non-parametric Mann–Whitney *U* test was used in most cases. For this reason, apart from the mean values and the standard deviation (SD), the median values, min. and max. and quartiles are also given. Statistically significant differences between the groups are also presented in Figs. 1S, 2S, 3S, 4S, 5S, 6S, 7S (as the supplementary files; online resource). Due to the large dispersion of the measurement results of some variables, two figures were made alternately: the cytokine concentration on the (vertical) axis on the real scale (Fig. 1S, 2S, 4S; online resource) and the cytokine concentration on the (vertical) axis on the logarithmic scale, based on the logarithm equal to 10, which clearly improves the legibility of their reception (Fig. 3S, 5S, 6S, 7S; online resource) (Tables 1 and 2).

**Table 1:** Cytokine levels in blood of healthy subjects (C)

Control group							
Cytokines (pg/ml)	*mean*	*median*	*lower* *quartile*	*upper* *quartile*	*standard* *deviation*	*lowest* *value*	*highest* *value*
IL-1β	0.114	0.103	0.096	0.114	0.038	0.088	0.248
IL-4	6.674	2.1	0.514	9.35	8.65	0.451	26.5
IL-6	11.657	7.88	3.71	18.23	10.242	0.811	36.4
IL-10	1.255	1.198	1.163	1.258	0.333	0.906	2.781
IL-17A	0.66	0.579	0.498	0.645	0.288	0.459	1.84
IL-17F	1.31	0.924	0.864	1.42	0.822	0.805	3.69
IL-21	1.454	1.432	1.362	1.526	0.14	1.245	1.847
IL-22	1.758	1.737	1.648	1.836	0.145	1.53	2.053
IL-23	10.217	2.114	1.992	2.568	20.767	1.572	76.05
IL-25	0.521	0.422	0.381	0.455	0.342	0.348	1.87
IL-31	82.838	79.77	34.92	119.41	57.962	8.8	221.09
IL-33	55.269	26.44	10.38	70.48	65.62	1.663	248.96
IFN-γ	3.17	3.15	2.943	3.317	0.328	2.675	3.906
TNF-α	6.856	7.07	5.66	8.19	1.846	3.09	11.28
sCD40L	297.72	291.78	130.38	422.69	206.11	16.7	744.65
TGF-β1	246652	257003	166874	319372	142148	905	562376

**Table 2:** Cytokine levels in blood of patients with pernicious anemia (PA)

PA group							
Cytokines (pg/ml)	*mean*	*median*	*lower* *quartile*	*upper* *quartile*	*standard* *deviation*	*lowest* *value*	*highest* *value*
IL-1β	0.101	0.098	0.093	0.105	0.011	0.084	0.124
IL-4	1.242	0.493	0.459	1.16	2.226	0.16	12.36
IL-6	14.586	10.31	2.69	15.39	18.119	0.828	87.99
IL-10	1.869	1.267	1.198	1.345	1.847	1.068	9.06
IL-17A	0.552	0.524	0.484	0.586	0.128	0.407	1.165
IL-17F	1.055	0.894	0.857	0.954	0.561	0.805	3.34
IL-21	1.449	1.456	1.374	1.479	0.106	1.245	1.761
IL-22	1.691	1.638	1.559	1.697	0.304	1.441	3.24
IL-23	2.069	1.948	1.815	2.147	0.549	1.494	4.828
IL-25	0.427	0.414	0.397	0.439	0.09	0.364	0.91
IL-31	84.836	87.965	62.33	104.18	33.364	13.14	164.25
IL-33	8.457	1.692	1.529	5.22	13.915	1.259	52.83
IFN-γ	3.193	3.21	2.996	3.371	0.208	2.889	3.638
TNF-α	7.862	6.945	5.52	9.51	3.485	2.65	17.15
sCD40L	214.23	213.74	154.87	273.4	102.28	35.37	551.92
TGF-β1	199898	194283	132080	242820	91338	45230	423466

## Results

### Immune responses in PA (Table 3)

**Table 3:** The comparison of blood cytokine levels between patients with pernicious anemia (PA) and healthy subjects (C)

Cytokines (pg/ml)	PA group	Control group	*p* value
	median / mean ± SD	median / mean ± SD	
Th17-related immune response			
IL-17A	0.524 / 0.552 ± 0.128	0.579 / 0.660 ± 0.288	*0.0356
IL-17F	0.894 / 1.055 ± 0.561	0.924 / 1.31 ± 0.822	0.2376
IL-21	1.456 / 1.449 ± 0.106	1.432 / 1.454 ± 0.14	0.8584
IL-22	1.638 / 1.691 ± 0.304	1.737 / 1.758 ± 0.145	*0.0042
IL-6	10.31 / 14.586 ± 18.119	7.88 / 11.657 ± 10.242	0.8240
IL-1β	0.098 / 0.101 ± 0.011	0.103 / 0.114 ± 0.038	0.1289
IL-23	1.948 / 2.069 ± 0.549	2.114 / 10.217 ± 20.767	*0.0185
Treg-related immune response			
IL-10	1.267 / 1.869 ± 1.847	1.198 / 1.255 ± 0.333	*0.0164
TGF-β1	194283 / 199898 ± 91338	257003 / 246652 ± 142148	0.1299
Th1-related immune response			
IFN-γ	3.21 / 3.193 ± 0.208	3.15 / 3.17 ± 0.328	0.7478
TNF-α	6.945 / 7.862 ± 3.485	7.07 / 6.856 ± 1.846	0.3946
sCD40L	213.74 / 214.23 ± 102.28	291.78 / 297.72 ± 206.11	*0.0452
Th2-related immune response			
IL-4	0.493 / 1.242 ± 2.226	2.1 / 6.674 ± 8.65	*0.0001
IL-21	1.456 / 1.449 ± 0.106	1.432 / 1.454 ± 0.14	0.8584
IL-25	0.414 / 0.427 ± 0.09	0.422 / 0.521 ± 0.342	0.8002
IL-31	87.965 / 84.836 ± 33.364	79.77 / 82.838 ± 57.962	0.5651
IL-33	1.692 / 8.457 ± 13.915	26.44 / 55.269 ± 65.62	*0.0000
Tfh cells activity			
IL-21	1.456 / 1.449 ± 0.106	1.432 / 1.454 ± 0.14	0.8584
Th22 cells activity			
IL-22	1.638 / 1.691 ± 0.304	1.737 / 1.758 ± 0.145	*0.0042

^*^p < 0.05 indicates statistical significance; SD, standard deviation

IFN‐γ, Interferon‐γ; IL – interleukin; PA, Pernicious Anemia; sCD40L, ligand for the TNF-α receptor; Tfh, T follicular helper cell; TNF‐α, Tumor necrosis factor‐α; TGF-β1, Transforming Growth Factor-β1; Treg, Regulatory T cell

The analyzed Th17-related immune response, indicated by the activity of the cytokines produced by Th17 cells (such as IL-17A, IL-17F, IL-21, IL-22, IL-6), manifested in several ways. On the one hand, the level of IL-17A in the blood of PA patients was lower than in healthy controls (Fig. 1S; online resource), while the level of IL-17F was similar. On the other hand, the level of IL-22 in patients with PA was lower than in healthy individuals (Fig. 2S; online resource). Both IL-6 and IL-21 levels did not differ in PA patients and in healthy controls.

The Treg-related immune response, indicated by the activity of cytokines such as IL-10, TGF-β, presented itself by the fact that the level of IL-10 in the blood of PA patients was higher than in healthy controls (Fig. 3S; online resource), whereas the TGF-β1 levels did not differ between PA patients and healthy individuals.

The Th1-related immune response has been examined in relation with the activity of cytokines produced by Th1 cells, such as IFN-γ, TNF-α. Neither IFN-γ, nor TNF-α blood level differed between PA patients and healthy controls, although the level of sCD40L (CD154; TNF-α receptor ligand) was lower in PA patients than in healthy individuals (Fig. 4S; online resource).

The Th2-related immune response, indicated by the activity of cytokines such as IL-4, IL-21, IL-25, IL-31, IL-33, manifested in the level of IL-4 and IL-33 in the blood of PA patients being clearly lower than in healthy controls (Fig. 5S, 6S; online resource), while the concentration of IL-21, IL-25 and IL-31 being similar in PA and C groups.

The activity of Tfh (follicular helper) cells was indicated by the secretion of IL-21 by these cells and it did not differ between PA patients and healthy controls.

Th22 cells activity, indicated by the secretion of IL-22, was lower in patients with PA than in healthy individuals (Fig. 2S; online resource).

### Immunity imbalance in PA (Table 3)

The patients with PA develop a Th17/Treg imbalance, manifested as a decrease in the amount of IL-17A and IL-22 (produced by Th17 cells) versus an increase in the amount of IL-10 (produced by Treg cells) in the bloodstream. A number of the other cytokines tested by us, produced by both Th17 cells and Treg cells, remains unchanged in the PA patients. Among the cytokines regulating the differentiation of Th17 cells (IL-1β, IL-6, IL-21, IL-23 [29, 41], reduction in the amount of IL-23 in the blood was observed in the patients with PA (Fig. 7; online resource). It is therefore believed that this decrease in IL-23 secretion entails a decrease in the activity of Th17 cells in the secretion of IL-17A and IL-22 (Fig. 1S, 2S; online resource). However, we did not observe any changes in the amount of TGF-β1 involved in the differentiation of Treg cells, so the increase in IL-10 secretion in PA (Fig. 3S; online resource) occurs through a different mechanism.

The patients with PA also develop a Th1/Th2 imbalance, marked by a decrease in the amount of IL-4 and IL-33 (produced by Th2 cells) (Fig. 5S, 6S; online resource), which in the presence of unchanged amounts of IFN-γ and TNF-α (produced by Th1 cells) indicates a quantitative advantage of the cytokines participating in Th1-related immune response over the cytokines participating in Th2-related immune response. Serum level of the other cytokines tested by us, produced by both Th1 cells and Th2 cells, remains unchanged in the PA patients. Among the cytokines regulating the differentiation of Th2 cells, reduction in the amount of IL-4 (we did not measure IL-2) in the blood was observed in patients with PA, which could entail a decrease in the activity of Th2 cells in the production of IL-4 and IL-33 (Fig. 5S, 6S; online resource). However, we did not observe any changes in the amount of IFN-γ participating in the differentiation of Th1 cells (we did not exam the level of IL-12 and IL-2).

Thirdly, the patients with PA develop a Th17/Th1 imbalance in a form of a decrease in the amount of IL-17A and IL-22 (produced by Th17 cells) in the bloodstream (Fig. 1S, 2S; online resource). In the presence of an unchanged amount of IFN-γ and TNF-α (produced by Th1 cells), this could indicate a quantitative advantage of the cytokines participating in Th1-related immune response over the cytokines participating in Th17-related immune response.

## Discussion

Our study results indicate that T lymphocytes and their specific cytokines play a role in the course of PA. The observed changes may indicate the immune response to PA or be an element of the PA pathomechanism. The role and effects of specific cytokines produced by T cell subsets (such as Th1, Th2, and newly discovered Th17, Treg, Tfh, or Th22) are diverse, depending on interactions with other cytokines, distinct signaling pathways, phase of the disease, or etiological factor. It is generally recognized that T lymphocytes and their secretory cytokines play vital role in modulation of the immune response against intracellular (Th1) and extracellular (Th2) antigens. However, at the same time a change in the activity of T lymphocytes may also lead to breakdown of immune tolerance, maintenance and amplification of the autoimmune response and lymphocytic infiltration in certain diseases, adverse changes in the morphology and function of tissues and organs, as well as to ending or, on the contrary, maintaining the remission of the disease. It seems essential to understand the pathomechanism of PA in all the above aspects.

The immunity equilibrium of the immune cells, such as the Th1/Th2, the Th17/Treg, and the Th17/Th1 balance is necessary for the maintenance of the immune homeostasis. If the balance of the T cells subsets is damaged, the autoimmune response becomes enhanced which leads to autoimmune diseases. Indeed, both the Th1/Th2 and the Th17/Treg dichotomies are involved in the pathomechanism of autoimmune diseases [9, 28, 29, 39, 40, 42–46]. In our study, we showed that patients suffering from PA develop the Th1/Th2 imbalance with a quantitative advantage of cytokines participating in Th1-related immune response, the Th17/Treg imbalance with a quantitative advantage of cytokines participating in Treg-related response, as well as the Th17/Th1 imbalance with a quantitative predominance of cytokines participating in Th1-related immune response. Are the Th1-related imbalances that we have observed surprising? Could they be reversed in order to restore the immune homeostasis? The review of the literature gives the impression that in autoimmune diseases the increase in Th1-related immune response derives from the increase in the secretion of specific cytokines. Meanwhile, in PA, the advantage of Th1 cells in the Th1/Th2 and the Th17/Th1 systems is also a result of the lower activity of the latter component. The cytokines secreted by Th1 lymphocytes have a significant pro-inflammatory effect, which clearly influences the development of autoimmune diseases. For instance, IFN-γ and TNF-α are ones of the major cytokines in inflammation [7, 9]. Proton pump is the target autoantigen of Th1-type cytotoxic T cells in autoimmune gastritis [47]. As for the Th17/Treg correlation, the activity of both systems and their mutual influence are the basis for the proper functioning of the immune system. Thus, their imbalance seems to be crucial in the development of autoimmune diseases [9, 28, 39, 48]. Patients with thyroid-associated orbitopathy (TAO) are characterized by a low number of circulating Treg cells among peripheral blood mononuclear cells (PBMCs) with high CD4/CD8 ratios and abnormal cytokine expression. In vitro, a 24-h incubation of TAO patients’ PBMCs together with rabbit anti-thymocyte globulin substantially enhanced the expression of Treg cell markers FoxP3 and CD3^+^ CD4^+^ CD25^+^ CD127^low^ [49]. At the same time, after administration of anti-thymocyte globulin, a long-term remission of steroid-resistant TAO was noted [50]. The Treg/Th17 imbalance with an orientation to Th17 was confirmed in animal as well as human models of autoimmune diseases [9, 25, 26, 39, 43, 48, 51]. In PA, the Th17/Treg imbalance is also observed, although this time in favor of Treg cytokines. Such observation may indicate an adaptive response of the immune system in the course of PA, as IL-10 and TGF-β1 demonstrate an immunosuppressive function [52]. CD4 + Foxp3 + Treg cells maintain immune homeostasis and tolerance to autoantigens by suppressing effector T cells as well as innate cell activities. The Treg cells contribute to the negative control of various immune responses [53, 54], and prevent autoimmune responses by targeting T cells, dendritic cells, macrophages, mast cells and B cells [44]. This observation is also consistent with the results of studies on autoimmune thyroid disease [55], although some suggest that increased levels of Treg cells in patients with autoimmune thyroid disease are most likely unable to down-modulate the autoimmune response [56]. A possible explanation for this could be that Treg cells activity can change over the course of autoimmune disease [42]. It appears that the involvement of Treg cells in the development of autoimmune diseases should not be considered separately, but in relation to effector cells, mainly Th17 cells [9]. The current knowledge of the cytokine-mediated regulation is still incomplete.

Formerly, it was believed that the differentiation of a naive T cell is irreversible. However, recent studies show that already differentiated T lymphocytes may in fact turn into another subset, which may suggest that under certain conditions such interconversion in possible. CD4 + T cells can diverse into subsets with various functions, but these, in turn, can also amend their role after receiving certain stimuli [29, 40, 57–62]. Although the T cell plasticity might complicate research analysis, it would also offer a chance to restore immune homeostasis in patients suffering from autoimmune diseases. Though it may seem unlikely to repair tissues that have already been damaged by infiltration of lymphocytes, any slowing down of autoimmune processes by modifying the cytokine profile and reducing antibody production would be beneficial.

In a healthy individual, the undesirable CD4 + T cell responses are controlled by regulatory mechanisms mediated by CD4 + and CD8 + T cells. Human CD4 + T cells exposed to a Th17-differentiating milieu are more resistant to immune suppression by CD8 + T cells, compared to control Th0 cells. This effect is partially mediated by IL-17A, IL-17F and IL-17AF through their receptors on CD4 + T cells, but not by their influence on CD8 + T cells or antigen-presenting cells. IL-17 can act directly on non-Th17 effector CD4 + T cells, inducing suppressive resistance, which can be reversed by blockade of IL-1β, IL-6, or STAT3. Several studies reveal a role of IL-17A in mediating CD4-intrinsic immune resistance [14]. Does the decrease in IL-17A concentration in PA patients translate into a decline in the resistance to immunosuppression? If so, it would be a beneficial phenomenon, distinguishing PA from other autoimmune diseases in which an increase in IL-17A (as well as in IL-22, IL-23) activity is observed [63, 64].

A limitation of the study is related to quite frequent co-occurrence of PA and other autoimmune diseases (our previous research [5, 6]), which certainly modifies the activity of the "cytokine network" in tissues and blood. In order to reduce such a probability, we selected the study group and control group as described in the methodology. We also took into account other situation that might modify the test results. In recent literature has been demonstrated that AIG patients have higher serum IL-17A, IL-17F, and IL-21 levels and this doesn't occur in patients without AIG [65]. In our study, we observed that IL-17A levels in the blood of PA patients was lower than in healthy controls, while the level of IL-17F and IL-21 levels did not differ in PA patients and in healthy controls. It is possible that the difference is related to the degree of advancement of AIG. The patients in our study group presented advanced clinical manifestation of AIG i.e. PA.

## Conclusions

1. The subsets of T lymphocytes, such as Th1, Th2 and newly discovered Th17, Treg, or Th22, along with their specific cytokines play an important role in the patomechanism of PA.
2. Patients suffering from PA develop the Th17/Treg imbalance, marked by a decrease in the amount of IL-17A and IL-22 (produced by Th17 cells) versus an increase in the amount of IL-10 and an unchanged amount of TGF-β1 (produced by Treg cells) in blood stream, which may indicate an immunosuppressive (i. e., adaptive) response of the immune system.
3. Among the cytokines regulating the differentiation of Th17 cells (such as IL-1β, IL-6, IL-23), a reduction in the amount of IL-23 in the blood of PA patients was observed, which entails a decrease in Th17 cells activity in the secretion of IL-17A and IL-22. However, no changes in the amount of TGF-β1, involved in the differentiation of Treg cells, were observed, so the increase in IL-10 secretion in PA patients occurs through a different mechanism.
4. Patients with PA develop Th1/Th2 imbalance, marked by a decrease in the amount of IL-4 and IL-33 (produced by Th2 cells), which in the presence of unchanged amounts of IFN-γ and TNF-α (both produced by Th1 cells) indicates a quantitative advantage of cytokines participating in Th1-related immune response over cytokines participating in Th2-related immune response and confirms the known influence of Th1 cells on the development of autoimmune diseases.

### Supplementary information

Below is the link to the electronic supplementary material.

Supplementary file1 (PDF 75 KB)

Supplementary file2 (PDF 77 KB)

Supplementary file3 (PDF 74 KB)

Supplementary file4 (PDF 88 KB)

Supplementary file5 (PDF 75 KB)

Supplementary file6 (PDF 75 KB)

Supplementary file7 (PDF 72 KB)

## Data Availability

The datasets generated during and/or analyzed during the current study are available from the corresponding author on reasonable request.

## Abbreviations

IFN‐γ: Interferon‐γ
IL: Interleukin
PA: Pernicious Anemia
TGF-β: Transforming Growth Factor-β
Th: T helper
Treg: Regulatory T cell
TNF‐α: Tumor necrosis factor‐α

## References

[1] Schwartz M (1967). Antibody to intrinsic factor. Scand J Clin Lab Invest Suppl.

[2] Rojas Hernandez CM, Oo TH (2015). Advances in mechanisms, diagnosis, and treatment of pernicious anemia. Discov Med.

[3] Watanabe S, Ide N, Ogawara H, Yokohama A, Mitsui T, Handa H (2015). High percentage of regulatory T cells before and after vitamin B12 treatment in patients with pernicious anemia. Acta Haematol.

[4] Rusak E, Chobot A, Krzywicka A, Wenzlau J (2016). Anti-parietal cell antibodies – diagnostic significance. Adv Med Sci.

[5] Morawiec-Szymonik E, Foltyn W, Marek B, Kos-Kudła B, Kajdaniuk D (2019). Pernicious anaemia and endocrine glands antibodies. Endokrynol Pol.

[6] Morawiec-Szymonik E, Foltyn W, Marek B, Głogowska-Szeląg J, Kos-Kudła B, Kajdaniuk D (2020). Antibodies involved in the development of pernicious anemia and other autoimmune diseases. Pol Arch Intern Med.

[7] Nowis D, Wańkowicz-Kalińska A, Winiarska M, Gołąb J, Jakóbisiak M, Lasek W, Stokłosa T (2019). Zjawiska autoimmunizacyjne. Immunologia.

[8] Decmann A, Tőke J, Csöregh É, Gáspárdy G, Somogyi A (2021). Type 3 autoimmune polyglandular syndrome with multiple genetic alterations in a young male patient with type 1 diabetes mellitus. Endokrynol Pol.

[9] Janyga S, Marek B, Kajdaniuk D, Ogrodowczyk-Bobik M, Urbanek A, Bułdak Ł (2021). CD4+ cells in autoimmune thyroid disease. Endokrynol Pol.

[10] Chen D (2008). TH17 cells in development: an updated view of their molecular identity and genetic programming. Nat Rev Immunol.

[11] Harrington LE, Hatton RD, Mangan PR, Turner H, Murphy TL, Murphy KM (2005). Interleukin 17-producing CD4+ effector T cells develop via a lineage distinct from the T helper type 1 and 2 lineages. Nat Immunol.

[12] Langrish CL, Chen Y, Blumenschein WM, Mattson J, Basham B, Sedgwick JD (2005). IL-23 drives a pathogenic T cell population that induces autoimmune inflammation. J Exp Med.

[13] Waite JC, Skokos D (2012). Th17 response and inflammatory autoimmune diseases. Int J Inflam.

[14] Crawford MP, Sinha S, Renavikar PS, Borcherding N, Karandikar NJ (2020). CD4 T cell-intrinsic role for the T helper 17 signature cytokine IL-17: Effector resistance to immune suppression. Proc Natl Acad Sci U S A.

[15] Gołąb J, Jakóbisiak M, Firczuk M, Gołąb J, Jakóbisiak M, Lasek W, Stokłosa T (2019). Cytokiny. Immunologia.

[16] Yang L, Anderson DE, Baecher-Allan C, Hastings WD, Bettelli E, Oukka M (2008). IL-21 and TGF-β are required for differentiation of human T(H)17 cells. Nature.

[17] Chung Y, Chang SH, Martinez GJ, Yang XO, Nurieva R, Soon Kang H (2009). Critical regulation of early Th17 cell differentiation by interleukin-1 signaling. Immunity.

[18] Sutton CE, Lalor SJ, Sweeney CM, Brereton CF, Lavelle EC, Mills KHG (2009). Interleukin-1 and IL-23 induce innate IL-17 production from γδ T cells, amplifying Th17 responses and autoimmunity. Immunity.

[19] Hirahara K, Ghoreschi K, Laurence A, Yang X-P, Kanno Y, O’Shea JJ (2010). Signal transduction pathways and transcriptional regulation in Th17 cell differentiation. Cytokine Growth Factor Rev.

[20] Wu B, Wan Y (2020). Molecular control of pathogenic Th17 cells in autoimmune diseases. Int Immunopharmacol.

[21] Das J, Ren G, Zhang L, Roberts AI, Zhao X, Bothwell ALM (2009). Transforming growth factor β is dispensable for the molecular orchestration of Th17 cell differentiation. J Exp Med.

[22] Ghoreschi K, Laurence A, Yang XP, Tato CM, McGeachy MJ, Konkel JE (2010). Generation of pathogenic T(H)17 cells in the absence of TGF-β signalling. Nature.

[23] Long D, Chen Y, Wu H, Zhao M, Lu Q (2019). Clinical significance and immunobiology of IL-21 in autoimmunity. J Autoimmun.

[24] Nurieva R, Yang XO, Martinez G, Zhang Y, Panopoulos AD, Ma L (2007). Essential autocrine regulation by IL-21 in the generation of inflammatory T cells. Nature.

[25] Li C, Yuan J, Zhu YF, Yang XJ, Wang Q, Xu J (2016). Imbalance of Th17/Treg in different subtypes of autoimmune thyroid diseases. Cell Physiol Biochem.

[26] Vitales-Noyola M, Ramos-Levi AM, Martínez-Hernández R, Serrano-Somavilla A, Sampedro-Nuñez M, González-Amaro R (2017). Pathogenic Th17 and Th22 cells are increased in patients with autoimmune thyroid disorders. Endocrine.

[27] Zake T, Skuja S, Kalere I, Konrade I, Groma V (2019). Upregulated tissue expression of T helper (Th) 17 pathogenic interleukin (IL)-23 and IL-1β in Hashimoto's thyroiditis but not in Graves' disease. Endocr J.

[28] Le Menn G, Jabłońska A, Chen Z (2022). The effects of post-translational modifications on Th17/Treg cell differentiation. Biochim Biophys Acta Mol Cell Res.

[29] Li Q, Wang B, Mu K, Zhang J-A (2019). The pathogenesis of thyroid autoimmune diseases: New T lymphocytes - Cytokines circuits beyond the Th1-Th2 paradigm. J Cell Physiol.

[30] Hendrawan K, Khoo MLM, Visweswaran M, Massey JC, Withers B, Sutton I (2022). Long-Term Suppression of Circulating Proinflammatory Cytokines in Multiple Sclerosis Patients Following Autologous Haematopoietic Stem Cell Transplantation. Front Immunol.

[31] Singh K, Rathore U, Rai MK, Behera MR, Jain N, Ora M (2022). Novel Th17 lymphocyte populations, Th17.1 and PD1+Th17, are increased in Takayasu arteritis, and both Th17 and Th17.1 sub-populations associate with active disease. J Inflamm Res.

[32] Nakase H, Sato N, Mizuno N, Ikawa Y (2022). The influence of cytokines on the complex pathology of ulcerative colitis. Autoimmun Rev.

[33] Fakhfakh R, Zian Z, Elloumi N, Abida O, Bouallegui E, Houssaini H (2022). Th17 and Th1 cells in systemic lupus erythematosus with focus on lupus nephritis. Immunol Res.

[34] Troilo A, Grassi A, Petrone L, Cianchi F, Benagiano M, Bella CD (2019). Intrinsic factor recognition promotes T helper 17/T helper 1 autoimmune gastric inflammation in patients with pernicious anemia. Oncotarget.

[35] Dobrakowski M, Boroń M, Czuba ZP, Kasperczyk A, Machoń-Grecka A, Kasperczyk S (2016). Cytokines related to three major types of cell-mediated immunity in short- and long-term exposures to lead compounds. J Immunotoxicol.

[36] Grudzińska E, Lekstan A, Szliszka E, Czuba ZP (2018). Cytokines produced by lymphocytes in the incompetent great saphenous vein. Mediators Inflamm.

[37] Grudzińska E, Grzegorczyn S, Czuba ZP (2019). Chemokines and growth factors produced by lymphocytes in the incompetent great saphenous vein. Mediators Inflamm.

[38] Li ZD, Mo XJ, Yan S, Wang D, Xu B, Guo J (2020). Multiplex cytokine and antibody profile in cystic echinococcosis patients during a three-year follow-up in reference to the cyst stages. Parasit Vectors.

[39] Jadidi-Niaragh F, Mirshafiey A (2012). The deviated balance between regulatory T cell and Th17 in autoimmunity. Immunopharmacol Immunotoxicol.

[40] Chen L, Ge B, Casale FP, Vasquez L, Kwan T, Garrido-Martín D (2016). Genetic drivers of epigenetic and transcriptional variation in human immune cells. Cell.

[41] Shao S, Yu X, Shen L (2018). Autoimmune thyroid diseases and Th17/Treg lymphocytes. Life Sci.

[42] González-Amaro R, Marazuela M (2016). T regulatory (Treg) and T helper 17 (Th17) lymphocytes in thyroid autoimmunity. Endocrine.

[43] Qin J, Zhou J, Fan C, Zhao N, Liu Y, Wang S (2017). Increased circulating Th17 but decreased CD4+Foxp3+ Treg and CD19+CD1dhiCD5+ Breg subsets in new-onset Graves' disease. Biomed Res Int.

[44] Chen Z, Wang Y, Ding X, Zhang M, He M, Zhao Y (2020). The proportion of peripheral blood Tregs among the CD4+ T cells of autoimmune thyroid disease patients: a meta-analysis. Endocr J.

[45] Aarts J, van Caam A, Chen X, Marijnissen RJ, Helsen MM, Walgreen B (2022). Local inhibition of TGF-β1 signaling improves Th17/Treg balance but not joint pathology during experimental arthritis. Sci Rep.

[46] Honda M, Segawa T, Ishikawa K, Maeda M, Saito Y, Kon S (2022). Nephronectin influences EAE development by regulating the Th17/Treg balance via reactive oxygen species. Am J Physiol Cell Physiol.

[47] D'Elios MM, Bergman MP, Azzurri A, Amedei A, Benagiano M, De Pont JJ (2001). H(+), K(+)-atpase (proton pump) is the target autoantigen of Th1-type cytotoxic T cells in autoimmune gastritis. Gastroenterology.

[48] Yuan Q, Zhao Y, Zhu X, Liu X (2017). Low regulatory T cell and high IL-17 mRNA expression in a mouse Graves' disease model. J Endocrinol Invest.

[49] Kahaly GJ, Shimony O, Gellman YN, Lytton SD, Eshkar-Sebban L, Rosenblum N (2011). Regulatory T-cells in Graves' orbitopathy: baseline findings and immunomodulation by anti-T lymphocyte globulin. J Clin Endocrinol Metab.

[50] Świerkot M, Kulawik G, Sarnat-Kucharczyk M, Jagoda K, Mrukwa-Kominek E, Chudek J (2020). Long-term remission of steroid-resistant Graves' orbitopathy after administration of anti-thymocyte globulin. Endokrynol Pol.

[51] Vitales-Noyola M, Serrano-Somavilla A, Martínez-Hernández R, Sampedro-Nuñez M, Ramos-Levi AM, González-Amaro R (2018). Patients with autoimmune thyroiditis show diminished levels and defective suppressive function of Tr1 regulatory lymphocytes. J Clin Endocrinol Metab.

[52] Kajdaniuk D, Marek B, Borgiel-Marek H, Kos-Kudła B (2013). Transforming growth factor β1 (TGFβ1) in physiology and pathology. Endokrynol Pol.

[53] Sakaguchi S (2004). Naturally arising CD4+ regulatory t cells for immunologic self-tolerance and negative control of immune responses. Annu Rev Immunol.

[54] Harakal J, Rival C, Qiao H, Tung KS (2016). Regulatory T cells control Th2-dominant murine autoimmune gastritis. J Immunol.

[55] Marazuela M, García-López MA, Figueroa-Vega N, de la Fuente H, Alvarado-Sánchez B, Monsiváis-Urenda A (2006). Regulatory T cells in human autoimmune thyroid disease. J Clin Endocrinol Metab.

[56] Rodríguez-Muñoz A, Vitales-Noyola M, Ramos-Levi A, Serrano-Somavilla A, González-Amaro R, Marazuela M (2016). Levels of regulatory T cells CD69(+)NKG2D(+)IL-10(+) are increased in patients with autoimmune thyroid disorders. Endocrine.

[57] Cosmi L, Maggi L, Santarlasci V, Liotta F, Annunziato F (2014). T helper cells plasticity in inflammation: T Helper Cells Plasticity in Inflammation. Cytometry A.

[58] Geginat J, Paroni M, Maglie S, Alfen JS, Kastirr I, Gruarin P (2014). Plasticity of human CD4 T cell subsets. Front Immunol.

[59] Ellmeier W (2015). Molecular control of CD4(+) T cell lineage plasticity and integrity. Int Immunopharmacol.

[60] Guéry L, Hugues S (2015). Th17 Cell Plasticity and Functions in Cancer Immunity. Biomed Res Int.

[61] DuPage M, Bluestone JA (2016). Harnessing the plasticity of CD4+T cells to treat immune-mediated disease. Nat Rev Immunol.

[62] Geginat J, Paroni M, Kastirr I, Larghi P, Pagani M, Abrignani S (2016). Reverse plasticity: TGF-beta and IL-6 induce Th1-to-Th17-cell transdifferentiation in the gut. Eur J Immunol.

[63] Peng D, Xu B, Wang Y, Guo H, Jiang Y (2013). A high frequency of circulating th22 and th17 cells in patients with new onset graves' disease. PLoS One.

[64] Ruggeri RM, Saitta S, Cristani M, Giovinazzo S, Tigano V, Trimarchi F (2014). Serum interleukin-23 (IL-23) is increased in Hashimoto's thyroiditis. Endocr J.

[65] Della Bella C, Antico A, Panozzo MP, Capitani N, Petrone L, Benagiano M (2022). Gastric Th17 Cells Specific for H+/K+-ATPase and Serum IL-17 Signature in Gastric Autoimmunity. Front Immunol.

