# Supplementary material for: Th17 cytokines and factors modulating their activity in patients with pernicious anemia
Source: Immunol Res. 2023 Jun 3;71(6):873–82. doi: 10.1007/s12026-023-09399-9 (PMC10667422; doi:10.1007/s12026-023-09399-9)
Supplement: Supplementary file 4 — Supplementary file4 (PDF 88 KB) [file 12026_2023_9399_MOESM4_ESM.pdf]

Immunologic Research

Th17 cytokines and factors modulating their activity in patients with pernicious anemia

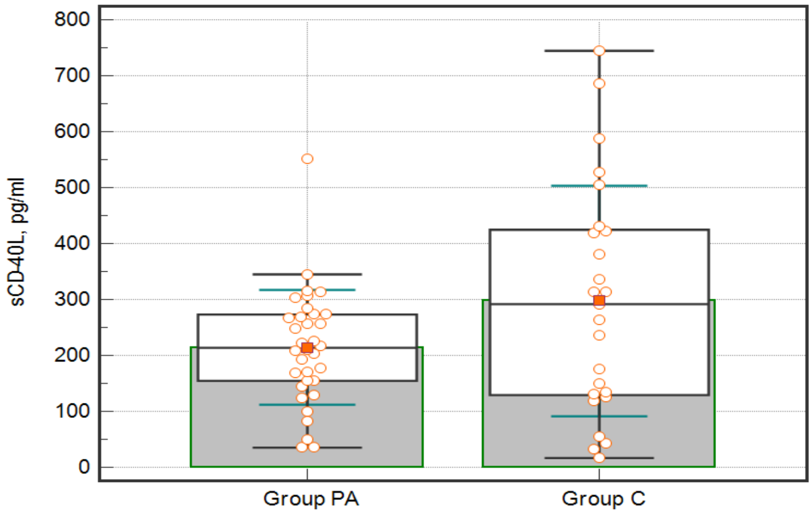

Fig. 4S The concentration of sCD-40L in the blood of PA patients and healthy controls (C)
The difference between the groups is statistically significant. The mean ±SD and also median, quartiles 25% and 75% are presented (Student *t* test).

[1]Dariusz Kajdaniuk*, [2]Wanda Foltyn, [3]Elżbieta Morawiec-Szymonik, [4]Zenon Czuba, [5]Ewa Szymonik, [2]Beata Kos-Kudła, [1]Bogdan Marek

[1]Department of Pathophysiology, Chair of Pathophysiology and Endocrinology, Medical University of Silesia, Katowice, Poland
[2]Department of Endocrinology and Neuroendocrine Tumors, Chair of Pathophysiology and Endocrinology, Medical University of Silesia, Katowice, Poland
[3]Department of Internal Medicine and Oncological Chemotherapy, Andrzej Mielęcki Independent Public Clinical Hospital, Katowice, Poland
[4]Department of Microbiology and Immunology, Medical University of Silesia, Katowice, Poland
[5]Department of Anesthesiology and Intensive Care, Stanislaw Szyszko Independent Public Clinical Hospital No. 1, Zabrze, Poland

*correspondending author:
dkajdaniuk@sum.edu.pl
